# Diversity of Multi-Drug Resistant Avian Pathogenic *Escherichia coli* (APEC) Causing Outbreaks of Colibacillosis in Broilers during 2012 in Spain

**DOI:** 10.1371/journal.pone.0143191

**Published:** 2015-11-23

**Authors:** Marc Solà-Ginés, Karla Cameron-Veas, Ignacio Badiola, Roser Dolz, Natalia Majó, Ghizlane Dahbi, Susana Viso, Azucena Mora, Jorge Blanco, Nuria Piedra-Carrasco, Juan José González-López, Lourdes Migura-Garcia

**Affiliations:** 1 Centre de Recerca en Sanitat Animal (CReSA)—Institut de Recerca i Tecnologia Agroalimentàries (IRTA), Campus UAB, Barcelona, Spain; 2 Departament de Sanitat i Anatomia Animals, Universitat Autònoma de Barcelona, Bellaterra (Cerdanyola del Vallés), Spain; 3 Laboratorio de Referencia de *E*. *coli*, Departamento de Microbioloxía e Parasitoloxía, Facultade de Veterinaria, Universidade de Santiago de Compostela, Lugo, Spain; 4 Servei de Microbiologia, Hospital Vall d’Hebron, Universitat Autònoma de Barcelona, Barcelona, Spain; The University of Hong Kong, CHINA

## Abstract

Avian pathogenic *Escherichia coli* (APEC) are the major cause of colibacillosis in poultry production. In this study, a total of 22 *E*. *coli* isolated from colibacillosis field cases and 10 avian faecal *E*. *coli* (AFEC) were analysed. All strains were characterised phenotypically by susceptibility testing and molecular typing methods such as pulsed-field gel electrophoresis (PFGE) and multi-locus sequence typing (MLST). The presence of 29 virulence genes associated to APEC and human extraintestinal pathogenic *E*. *coli* (ExPEC) was also evaluated. For cephalosporin resistant isolates, cephalosporin resistance genes, plasmid location and replicon typing was assessed. Avian isolates belonged to 26 O:H serotypes and 24 sequence types. Out of 22 APEC isolates, 91% contained the virulence genes predictors of APEC; *iutA*, *hlyF*, *iss*, *iroN* and *ompT*. Of all strains, 34% were considered ExPEC. PFGE analysis demonstrated a high degree of genetic polymorphism. All strains were multi-resistant, including those isolated from healthy animals. Eleven strains were resistant to cephalosporins; six contained *bla*_CTX-M-14_, two *bla*_SHV-12_, two *bla*_CMY-2_ and one *bla*_SHV-2_. Two strains harboured *qnrA*, and two *qnrA* together with *aac*(6’)-Ib-cr. Additionally, the emergent clone O25b:H4-B2-ST131 was isolated from a healthy animal which harboured *bla*_CMY-2_ and *qnrS* genes. Cephalosporin resistant genes were mainly associated to the presence of IncK replicons. This study demonstrates a very diverse population of multi-drug resistant *E*. *coli* containing a high number of virulent genes. The *E*. *coli* population among broilers is a reservoir of resistance and virulence-associated genes that could be transmitted into the community through the food chain. More epidemiological studies are necessary to identify clonal groups and resistance mechanisms with potential relevance to public health.

## Introduction

*Escherichia coli* is a bacterium widespread in the intestine of animals and humans, and a pathogen that can induce enteric and extraintestinal infections. In particular, avian pathogenic *E*. *coli* (APEC) is the main cause of colibacillosis in poultry farms; a syndrome associated to airsacculitis, perihepatitis, pericarditis, and sometimes fatal septicemia. APEC strains are responsible for the mortality of 3–4% of the animals in a farm, and for the reduction of 2–3% of egg production [1], resulting in an economic burden to the poultry industry [2]. In many cases, the fundamental cause of the disease remains unclear, since the infection with *E*. *coli* is associated to the presence of *Mycoplasma gallisepticum* or respiratory viruses, such as Newcastle virus or Infectious Bronchitis virus [3].

Several virulence genes are implicated in avian colibacillosis such as adhesins, toxins, anti-host defence factors, iron acquisition systems, autotransporters and the IbeA protein [4]. Subtractive hybridization studies have demonstrated sequence homology between specific DNA regions of APEC and human extraintestinal pathogenic *E*. *coli* (ExPEC) [5]. Additionally, the presence of similar virulence genes found in both, APEC and ExPEC strains, suggested that APEC strains may act as zoonotic pathogens and reservoir of virulence causing human infections [6–8]. According to Johnson *et al*. (2003), a strain could be considered ExPEC if exhibits two or more of the following virulence genes; *pap* (P fimbriae), *sfa/foc* (S/F1C fimbriae), *afa/dra* (Dr binding adhesins), *iutA* (aerobactin receptor), and *kpsM* II (group 2 capsule synthesis) [9]. ExPEC strains are more often derived from virulence-associated B2 and D phylogroups [10].

The successful treatment of avian colibacillosis caused by APEC strains mainly depends on the use of antimicrobials. However, increasing resistance to critically important antimicrobials, such as third-generation cephalosporins and fluoroquinolones, is nowadays common in *E*. *coli* from poultry origin [11]. These resistances can be transmitted to humans via the food supply [12, 13]. In particular, *E*. *coli* producing extended-spectrum beta-lactamases (ESBLs) and plasmid mediated AmpC beta-lactamases have increased considerably in the last years [14]. Normally, these genes are located on plasmids, and can be transferred by conjugation to other bacterial species [11]. Some of the virulence factors for APEC and ExPEC can also be harboured on plasmids. Particularly, ColV plasmids yield some virulence genes such as *hlyF*, *ompT*, *iss* and *cvaC* surrounding the replicon RepFIB [15].

Several studies have described APEC strains in the literature [16]. However, not many studies have combined extensive characterization at the serotype level, virulence-associated genes, molecular typing techniques, molecular determination of resistance mechanisms and mobile genetic elements involved in transfer of resistance. For this reason, the objective of this study was to discriminate and to perform such characterization of highly pathogenic *E*. *coli* causing outbreaks of colibacillosis in 13 different broiler farms throughout Spain, and compare them to avian faecal *E*. *coli* (AFEC) obtained from healthy animals. Additionally, the identification of clones more prone to cause disease has been assessed.

## Materials and Methods

### Isolation

**Fig 1:**
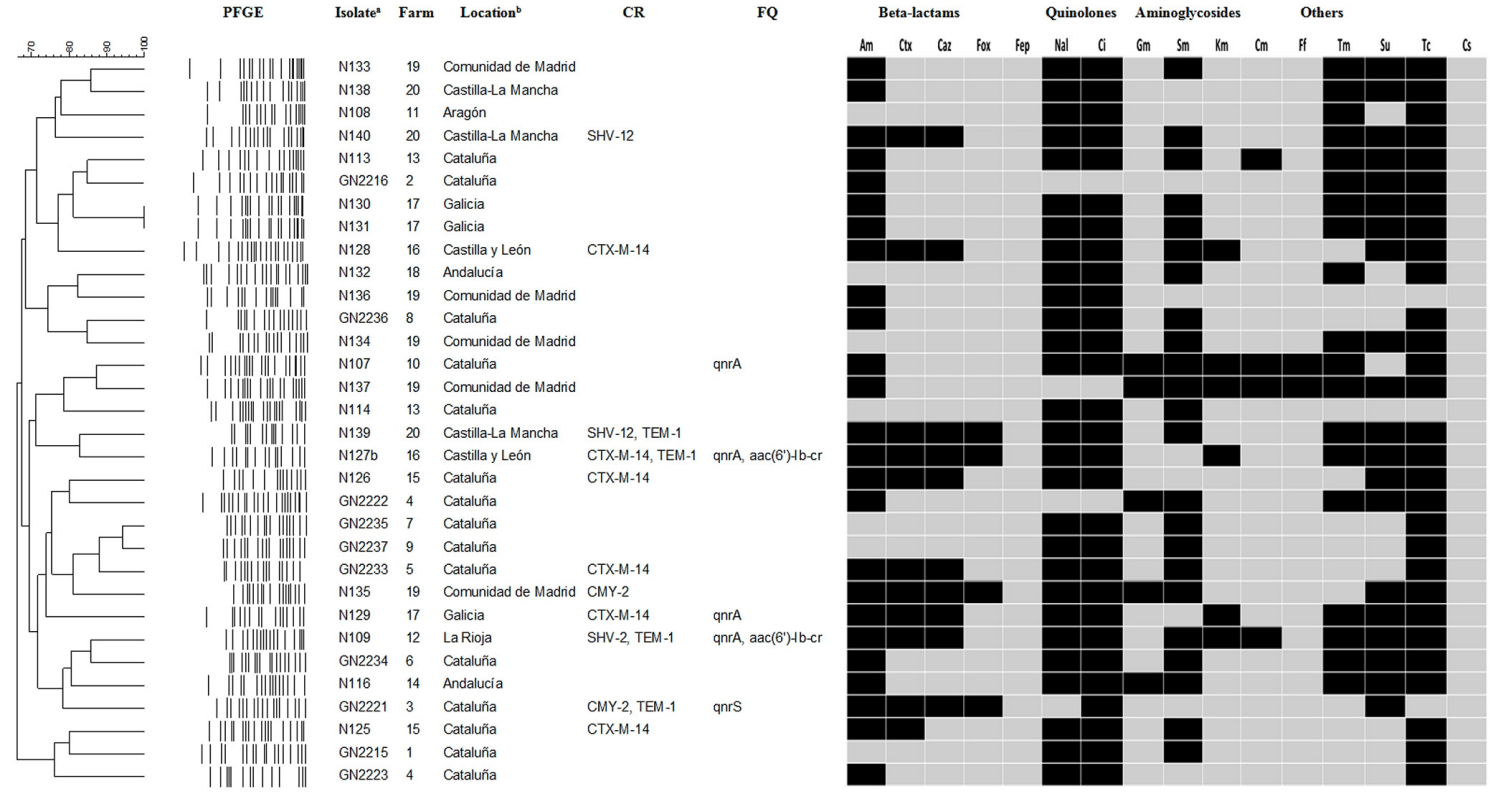
PFGE dendogram illustrating the phenotypic and genotypic relationship of the strains and the cephalosporin resistance genes. PFGE, pulsed-field gel electrophoresis; CR., cephalosporin resistance genes; FQ., flouoroquinolone resistance genes; Am: Ampicillin (WT≤8mg/L); Ctx: Cefotaxime (WT≤0.25mg/L); Caz: Ceftazidime (WT≤0.5mg/L); Fox: Cefoxitin (WT≤8mg/L); Fep: Cefepime (WT≤0.125mg/L); Nal: Nalidixic acid (WT≤16mg/L); Ci: Ciprofloxacin (WT≤0.064mg/L); Gm: Gentamicin (WT≤2mg/L); Sm: Streptomycin (WT≤16mg/L); Km: Kanamycin (WT≤8mg/L);. Cm: Chloramphenicol (WT≤16mg/L); Ff: Florfenicol (WT≤16mg/L); Tm: Trimethoprim (WT≤2mg/L); Su: Sulphamethoxazole (WT≤64mg/L); Tc: Tetracycline (WT≤8mg/L); Cs: Colistin (WT≤2mg/L). ^a^ Isolates are divided in APEC (N) and AFEC (GN) strains. ^b^ Location of the strains is named in order to the different regions of Spain where farms were localized.

A total of 22 tissue swabs of culled-animals affected with colibacillosis arrived to the laboratory between January and March 2012. The samples were obtained as part of routine care. Samples were taken from chickens already sacrificed for diagnostic purposes following the procedures according to the requirements of the Ethics Committee of Animal and Human Experimentation of the Universitat Autònoma de Barcelona (Permit Number DMAH-4239 that specifically permits euthanasia of chickens). Animals were euthanized using intravenous sodium pentobarbital (100 mg/kg, Dolethal, Vétoquinol, Cedex, France) in the wing vein. The method to sacrifice the animals follows the welfare rules stated in the European Directive 86/609/CEE. None of the authors of this manuscript were involved in manipulating or sacrificing the chickens. All the samples were collected from clinical cases submitted to the Diagnostic Service of the Veterinary School of the Universitat Autònoma de Barcelona. Swabs were taken from 13 broiler farms located in nine different regions of Spain (Fig 1). Ten *E*. *coli* strains isolated from faeces of healthy animals collected in nine farms were also included in the study. The samples were plated onto MacConkey agar and incubated overnight at 37°C. Three lactose-positive colonies for each plate were selected and confirmed to be *E*. *coli* by PCR [17]. Subsequently, one representative was selected for further studies.

### Serotyping

Determination of O and H antigens was carried out using the method previously described by Guinée *et al*. with all available O (O1 to O181) and H (H1 to H56) antisera [18]. Non-typeable isolates were denoted as ONT or HNT and non-motile isolates were denoted as HNM. All antisera were obtained and absorbed with the corresponding cross-reacting antigens to remove the nonspecific agglutinins. The O and H antisera were produced in the Laboratorio de Referencia de *E*. *coli* (LREC, Lugo, Spain). O25a and O25b subtypes were determined by PCR [19].

### Phylogeny, pulsed-field gel electrophoresis (PFGE) and multilocus sequence typing (MLST)

Isolates were separated in phylogroups (A, B1, B2, C, D, E or F) according to a method previously described [20, 21].

PFGE was performed as described elsewhere [22]. The results were analysed by Fingerprinting II Informatix software (Applied Maths, Sint-Martens-Latem, Belgium). PFGE-types were separated based on differences of at least one band in the restriction profiles. The analysis of the bands generated was carried out using the Dice coefficient and unweighted pair group method with arithmetic averages (optimization of 1.25% and position tolerance 1.25%).

MLST was carried out as previously described according to the protocol and primers specified on the *E*. *coli* MLST web site (http://mlst.ucc.ie/mlst/dbs/Ecoli) [23]. Sequences were analysed with Vector NTI advance 11 software (InforMax, Inc., Bethesda, MD).

### Detection of virulence-associated genes

All strains were tested by PCR for 29 ExPEC and APEC virulence-associated genes (Tables 1 and 2) [13, 24]. The genes described previously by Johnson *et al*. (2008) as the minimal predictors of APEC virulence; *iroN*, *ompT*, *hlyF*, *iutA* and *iss* were detected by a multiplex PCR [24]. Virulence scores were calculated for each isolate as the sum of all virulence-associated genes detected; *pap*, *sfa-foc* and *kpsM* II were counted only once.

**Table 1:** Distribution of virulence-associated gene profiles, phylogeny, serotyping and MLST results among all 32 strains.

	Isolate	Phylo.	Serotype	ST	Cplx	Virulence gene profiles^a^
**AFEC**						
	GN-2215	D	ONT:H4	ST117		***iss***, *fimH*, *fyuA*, *irp-2*, *cdtB*, *traT*, *malX*, *tsh*
	GN-2216	A	O53:H18	ST10	ST10 Cplx	***iss***, *fimH*, *traT*
	GN-2221	B2	O25b:H4	ST131		***iroN***, ***ompT***, ***iutA***, ***iss***, *fimH*, *cvaC*, *irp-2*, *kpsM II-K1*, *traT*, *ibeA*, *malX*, *usp*, *tsh*
	GN-2222	F	O83:HNT	ST648		***iroN***, ***ompT***, ***iutA***, ***iss***, *fimH*, *cvaC*, *astA*, *kspM II-K5*, *traT*, *malX*, *tsh*
	GN-2223	A	ONT:HNT	ST10	ST10 Cplx	*fimH*, *fyuA*, *irp-2*, *traT*
	GN-2233	A	O3:H26	ST165	ST165 Cplx	*fimH*, *fimAvMT78*, *traT*
	GN-2234	A	O2:H40	ST10	ST10 Cplx	***iroN***, ***ompT***, ***iss***, *fimH*, *fyuA*, *traT*
	GN-2235	A	O3:HNM	ST165	ST165 Cplx	*fimH*, *fimAvMT78*, *traT*
	GN-2236	B1	O127:H37	ST297		***ompT***, ***iutA***, ***iss***, *fimH*, *cvaC*, *traT*, *malX*, *tsh*
	GN-2237	A	O3:H26	ST189	ST165 Cplx	*fimH*, *fimAvMT78*, *traT*
**APEC**						* *
	N107	B1	ONT:H16	ST295		***iroN***, ***ompT***, ***hlyF***, ***iutA***, ***iss***, *fimH*, *cvaC*, *traT*, *tsh*
	N108	C	O78:H9	ST23	ST23 Cplx	***iroN***, ***ompT***, ***hlyF***, ***iutA***, ***iss***, *fimH*, *fyuA*, *astA*, *traT*, *tsh*
	N109	B1	ONT:H28	ST156	ST156 Cplx	***iroN***, ***ompT***, ***hlyF***, ***iutA***, ***iss***, *fimH*, *astA*, *irp-2*, *traT*
	N116	D	O25a:[H4]	ST624		***iroN***, ***ompT***, ***hlyF***, ***iutA***, ***iss***, *fimH*, *cvaC*, *kpsM III*, *traT*, *malX*
	N113	A	O5:H10	ST93	ST168 Cplx	***iroN***, ***ompT***, ***hlyF***, ***iutA***, ***iss***, *astA*, *kpsM II-K2*
	N114	A	O6:H16	ST48	ST10 Cplx	***iroN***, ***ompT***, ***hlyF***, ***iutA***, ***iss***, *fimH*, *fimAvMT78*, *kpsM III*, *traT*
	N125	A	O88:HNM	ST1137		***iroN***, ***ompT***, ***hlyF***, ***iutA***, ***iss***, *fimH*, *cvaC*, *astA*, *fimAvMT78*, *traT*
	N126	B1	ONT:H51	ST889		***iroN***, ***ompT***, ***hlyF***, ***iutA***, ***iss***, *fimH*, *cvaC*, *traT*, *tsh*
	N127b	D	ONT:H51	ST156	ST156 Cplx	***iroN***, ***ompT***, ***hlyF***, ***iutA***, ***iss***, *fimH*, *traT*, *tsh*
	N128	E	ONT:H27	ST350	ST350 Cplx	***iroN***, ***ompT***, ***hlyF***, ***iutA***, ***iss***, *fimH*, *cvaC*, *astA*, *irp-2*, *fimAvMT78*, *papEF*, *papG*, *traT*, *tsh*
	N129	B1	O15:H10	ST101	ST101 Cplx	***iroN***, ***ompT***, ***hlyF***, ***iutA***, ***iss***, *cvaC*, *astA*, *sfa/focDE*, *traT*, *tsh*
	N130	A	O5:H51	ST93	ST168 Cplx	***iroN***, ***ompT***, ***hlyF***, ***iutA***, ***iss***, *astA*, *kspM II-K2*
	N131	A	O5:H51	ST93	ST168 Cplx	***iroN***, ***ompT***, ***hlyF***, ***iutA***, ***iss***, *astA*, *kspM II-K2*
	N132	E	O102:H25	ST57	ST350 Cplx	***iroN***, ***ompT***, ***hlyF***, ***iutA***, ***iss***, *fimH*, *traT*, *tsh*
	N133	A	O5:H10	ST93	ST168 Cplx	***iroN***, ***ompT***, ***hlyF***, ***iutA***, ***iss***, *kpsM II-K2*
	N134	B1	O159:H28	ST539		***ompT***, ***hlyF***, ***iutA***, ***iss***, *fimH*, *irp-2*, *traT*
	N135	B2	O2:H1	ST429		***iroN***, ***ompT***, ***hlyF***, ***iutA***, ***iss***, *fimH*, *fyuA*, *cvaC*, *irp-2*, *kpsM II-K1*, *traT*, *ibeA*, *malX*, *usp*, *tsh*
	N136	D	ONT:H4	ST830		***iroN***, ***ompT***, ***hlyF***, ***iutA***, ***iss***, *fimH*, *fyuA*, *traT*, *malX*
	N137	D	O11:H15	ST3161		***iroN***, ***ompT***, ***hlyF***, ***iutA***, ***iss***, *fimH*, *cvaC*, *traT*
	N138	C	O78:H9	ST650	ST23 Cplx	***iroN***, ***ompT***, ***hlyF***, ***iutA***, ***iss***, *fimH*, *traT*
	N139	B1	O45:H8	ST533		***iroN***, ***ompT***, ***hlyF***, *fimH*, *astA*, *traT*, *malX*
	N140	E	O119:H27	ST350	ST350 Cplx	***iutA***, *fimH*, *astA*, *fimAvMT78*

Phylo, phylogroup; ST, sequence type; Cplx, clonal complex. Adhesins *fimH* (D-mannose-specific adhesin of type I fimbriae), *fimAvMT78* (FimA variant MT78 of type 1 fimbriae), *papEF* and *papG* (P fimbria subunits), and *sfa/focDE* (S fimbrial adhesin/putative F1C fimbrial adhesin); toxins *cdtB* (cytolethal distending toxin), *hlyF* (hemolysin F), and *astA* (EAST1, enteroaggregative E. coli heat-stable toxin); siderophores fyuA (yersiniabactin), *iutA* (aerobactin), *iroN* (novel catecholate siderophore receptor), and *irp-2* (iron repressible associated with yersiniabactin synthesis); protectins *kpsM* (groups II and III, specifically targeting the K1, K2 and K5 genes of group II capsules), *cvaC* (ColV, colicin V from serum resistance-associated plasmids), *iss* (surface exclusion serum survival protein), and *traT* (serum resistance); miscellaneous virulence genes *ompT* (protease), *ibeA* (invasion of brain endothelium), *malX* (PAI, pathogenicity island marker), and *usp* (uropathogenic-specific protein, bacteriocin).

^a^ Virulence-associated genes shown in boldface are the five genes characteristics of APEC strains.

**Table 2:** Distribution and characterization of virulence-associated genes and phylogroups of the 32 isolates.

Virulence gene (s)^a^	Total isolates (%) (n = 32)	APEC isolates (%) (n = 22)	AFEC isolates (%) (n = 10)	A (%) (n = 12)	B1 (%) (n = 7)	B2 (%) (n = 2)	D (%) (n = 5)	C, E and F (%) (n = 6)	B2/D (%) (n = 7)	A/B1 (%) (n = 19)	A/APEC (%) (n = 6)	A/AFEC (%) (n = 6)	*P* value^b^ APEC vs AFEC	*P* value^b^ A/APEC vs A/AFEC	*P* value^b^ B2/D vs A/B1
**Adhesins**															
*fimH*	28 (88%)	18 (82%)	10 (100%)	8 (67%)	7 (100%)	2 (100%)	5 (100%)	5 (83%)	7 (100%)	15 (79%)	2 (33%)	6 (100%)			
*fimAvMT78*	7 (22%)	4 (18%)	3 (30%)	5 (42%)	0	2 (100%)	0	2 (33%)	2 (29%)	5 (26%)	2 (33%)	3 (50%)			
*papEF*	1 (3%)	1 (5%)	0	0	0	0	0	1 (17%)	0	0	0	0			
*papG*	1 (3%)	1 (5%)	0	0	0	0	0	1 (17%)	0	0	0	0			
*sfa/focDE*	1 (3%)	1 (5%)	0	0	1 (14%)	0	0	0	0	1 (5%)	0	0			
*afa/draBC*	0	0	0	0	0	0	0	0	0	0	0	0			
Toxins															
*cnf1*	0	0	0	0	0	0	0	0	0	0	0	0			
*cdtB*	1 (3%)	0	1 (10%)	0	0	0	1 (20%)	0	1 (15%)	0	0	0			
*sat*	0	0	0	0	0	0	0	0	0	0	0	0			
*hlyA*	0	0	0	0	0	0	0	0	0	0	0	0			
hlyF*	22 (72%)	22 (100%)	0	6 (50%)	6 (86%)	1 (50%)	4 (80%)	4 (67%)	5 (71%)	12 (63%)	6 (100%)	0	<0.0001	0.0022	
*astA*	12 (38%)	11 (50%)	1 (10%)	5 (42%)	3 (43%)	0	1 (20%)	3 (50%)	0	8 (42%)	5 (83%)	0	0.0496	0.0152	
*tsh*	12 (38%)	8 (36%)	4 (40%)	0	4 (57%)	2 (100%)	2 (40%)	4 (67%)	4 (57%)	4 (21%)	0	0			
**Siderophores**															
*fyuA*	9 (28%)	5 (23%)	4 (40%)	2 (17%)	1 (14%)	2 (100%)	3 (60%)	1 (17%)	5 (71%)	3 (16%)	0	2 (33%)			0.0138
iutA*	24 (75%)	21 (95%)	2 (20%)	6 (50%)	6 (86%)	2 (100%)	4 (80%)	6 (100%)	6 (86%)	12 (63%)	6 (100%)	0	<0.0001	0.0022	
iroN*	24 (72%)	21 (95%)	3 (30%)	7 (58%)	6 (86%)	2 (100%)	4 (80%)	5 (83%)	6 (86%)	13 (68%)	6 (100%)	1 (17%)	0.0003	0.0152	
*irp-2*	8 (25%)	5 (23%)	3 (30%)	1 (8%)	3 (43%)	2 (100%)	1 (20%)	1 (17%)	3 (43%)	4 (21%)	0	1 (17%)			
**Protectins**															
*kpsM* II	7 (22%)	5 (23%)	2 (20%)	4 (33%)	0	2 (100%)	0	1 (17%)	2 (29%)	4 (21%)	4 (67%)	0			
*kpsM* II-K1	2 (6%)	1 (5%)	1 (10%)	0	0	2 (100%)	0	0	2 (29%)	0	0	0			
*kpsM* II-K2	4 (13%)	4 (18%)	0	4 (33%)	0	0	0	0	0	4 (2%)	4 (67%)	0			
*kpsM* II-K5	1 (3%)	0	1 (10%)	0	0	0	0	1 (17%)	0	0	0	0			
*kpsM* III	2 (6%)	2 (9%)	0	1 (8%)	0	0	1 (20%)	0	1 (15%)	1 (5%)	1 (17%)	0			
*cvaC*	11 (34%)	9 (41%)	3 (30%)	1 (8%)	4 (57%)	2 (100%)	2 (40%)	2 (33%)	4 (57%)	5 (26%)	1 (17%)	0			
iss*	26 (81%)	20 (91%)	6 (60%)	8 (67%)	7 (100%)	2 (100%)	5 (100%)	5 (83%)	7 (100%)	15 (79%)	6 (100%)	2 (33%)			
*traT*	27 (84%)	17 (77%)	10 (100%)	8 (67%)	7 (100%)	2 (100%)	5 (100%)	5 (83%)	7 (100%)	15 (79%)	2 (33%)	6 (100%)			
**Miscellaneous**															
ompT*	26 (78%)	22 (100%)	4 (40%)	7 (58%)	7 (100%)	2 (100%)	4 (80%)	3 (50%)	6 (86%)	14 (74%)	6 (100%)	1 (17%)	0.0002	0.0152	
*ibeA*	2 (6%)	1 (5%)	1 (10%)	0	0	2 (100%)	0	0	2 (29%)	0	0	0			
*malX*	8 (25%)	4 (18%)	4 (40%)	0	1 (14%)	2 (100%)	3 (60%)	1 (17%)	5 (71%)	1 (5%)	0	0			0.0018
*usp*	2 (6%)	1 (5%)	1 (10%)	0	0	2 (100%)	3 (60%)	0	5 (71%)	0	0	0			0.0003
**Mean (range) virulence score** ^c^	8.2 (3–16)	9.2 (5–16)	6.7 (3–13)	5.8 (3–11)	10 (8–13)	14.5 (13–16)	9.8 (8–11)	9.2 (5–14)	11.1 (8–16)	7.4 (3–13)	7.2 (5–11)	4.2 (3–6)	0.035	0.013	0.008

Adhesins *fimH* (D-mannose-specific adhesin of type I fimbriae), *fimAvMT78* (FimA variant MT78 of type 1 fimbriae), *papEF* and *papG* (P fimbria subunits), *sfa/focDE* (S fimbrial adhesin/putative F1C fimbrial adhesin), and *afa/draBC* (Dr antigen specific adhesin); toxins *cnf1* (cytotoxic necrotizing factor 1), *cdtB* (cytolethal distending toxin), *sat* (secreted autotransporter toxin), *hlyA* (α-hemolysin), *hlyF* (hemolysin F), and *astA* (EAST1, enteroaggregative E. coli heat-stable toxin); siderophores fyuA (yersiniabactin), *iutA* (aerobactin), *iroN* (novel catecholate siderophore receptor), and *irp-2* (iron repressible associated with yersiniabactin synthesis); protectins *kpsM* (groups II and III, specifically targeting the K1, K2 and K5 genes of group II capsules), *cvaC* (ColV, colicin V from serum resistance-associated plasmids), *iss* (surface exclusion serum survival protein), and *traT* (serum resistance); miscellaneous virulence genes *ompT* (protease), *ibeA* (invasion of brain endothelium), *malX* (PAI, pathogenicity island marker), and *usp* (uropathogenic-specific protein, bacteriocin).

^a^ Virulence-associated genes shown with asterisk are the five genes characteristics of APEC strains.

^b^ *P* values (by Fisher’s exact test) are shown where P<0.05.

^c^ The virulence score was the number of virulence genes detected, adjusted for multiple detection of the *pap*, *sfa* and *foc*, and *kpsM* II operons. Virulence scores were compared by use of the Mann-Whitney U test.

### Statistical analysis

Differences in the prevalence between different groups were determined by Fisher’s exact test as described before [25]. Virulence scores were compared by the use of Mann-Whitney U test. Statistical analyses were performed using GraphPad Prsim, version 3.1 software (GraphPad Software, Inc., San Diego, CA).

### Antimicrobial susceptibility testing

Disc diffusion was performed according to CLSI guidelines using the following discs (Oxoid, Basingstoke, UK): cefoxitin, 30 mg; cefepime, 30 mg; ceftazidime, 30 mg; cefotaxime, 30 mg; cefotaxime+clavulanic acid, 30+10 mg; and ceftazidime+clavulanic acid, 30+10 mg. The disc combinations of cefotaxime and cefotaxime/clavulanic acid, ceftazidime and ceftazidime/clavulanic acid were used for the identification of ESBLs; cefoxitin was used for the detection of AmpC-type beta-lactamase [26]. All isolates were susceptibility tested using a minimum inhibitory concentration (MIC)-based broth microdilution (VetMIC GN-mo, National Veterinary Institute, Uppsala, Sweden) as described before [25]. Isolates were considered to be wild type (WT) or non-WT based on epidemiological cut-off values according to EUCAST (http://www.eucast.org/).

### Resistance genes

All strains exhibiting resistance to third-generation cephalosporins (cefotaxime and ceftazidime) were tested by PCR methods for the presence of the *bla*_CTX-M_, *bla*_SHV_, *bla*_TEM_, *bla*_CMY-1_ and *bla*_CMY-2_ genes as described by Hasman *et al*. [27]. Detection of plasmid-mediated AmpC beta-lactamase genes was assessed by multiplex PCR [28]. Sequencing of both strands of amplicons was performed. The presence of the fluoroquinolone resistance genes *aac(6’)-Ib-cr*, *qnrA*, *qnrB*, *qnrS*, *qepA* and *oqxAB* was also assessed [29, 30].

### Plasmid DNA analysis

Isolates exhibiting resistance to cephalosporins were selected for plasmid characterization. Plasmid replicons tested were elected according to the presence of determinant resistance genes (HI1, HI2, I1, X, L/M, N, FIA, FIB, W, Y, P, FIC, A/C, T, FIIA and K) [31] and were identified using the PCR-based replicon typing method previously described [32, 33]. Plasmids detection and sizing was performed on all the isolates by S1-nuclease PFGE of total DNA [34]. Restriction fragments from S1-PFGE gels were transferred onto a positively charged nylon membrane and hybridised with specific probes for *bla*_CTX-M-14_, *bla*_TEM_, *bla*_SHV_, *bla*_CMY_ and for each replicon that was previously identified.

## Results

A total of 22 *E*. *coli* were recovered from 13 different farms distributed throughout Spain during 2012. Additionally 10 isolates from healthy animals collected in nine different farms were also included in the study to make a total of 32 *E*. *coli* isolates.

### Serotypes

A total of 16 different O serogroups, 15 flagellar H antigens and 26 different O:H serotypes were identified (Table 1). The most prevalent serotypes were: O3:H26, O5:H10, O5:H51 and O78:H9. Additionally, the emergent clone O25b:H4 was detected in a commensal isolate.

### Phylogeny, PFGE and MLST

Within the APEC strains (n = 22) the phylotyping identified six strains belonging to group A (27%), six to group B1 (27%), four to group D (18%), three to group E (14%), two to group C (9%) and one to group B2 (5%). For the commensal strains (n = 10), 60% belonged to phylogroup A, and the remaining 40% to B1, B2, D and F phylogroups (10% each) (Table 1).

*XbaI*-PFGE analysis showed a high degree of genetic polymorphism. A total of 31 different PFGE restriction profiles were identified among the 32 *E*. *coli* isolates (Fig 1). Only two isolates were epidemiologically related and belonged to the same farm.

Among the APEC subgroup (n = 22), MLST analyses identified 18 STs, while six STs were found among AFEC isolates (n = 10) (Table 1). Within the APEC strains, four belonged to the ST168 clonal complex (Cplx), three to the ST350 Cplx, two to ST156 Cplx, and two to the highly pathogenic ST23 Cplx (Table 1). Within the AFEC isolates, the most common Cplx was ST10 (n = 3), followed by ST165 Cplx (n = 3) (Table 1). The emergent pandemic multirresistant clone O25b:H4-B2-ST131 was found among the AFEC isolates.

### Detection of virulence-associated genes

The prevalence of 29 virulence-associated genes is shown in Tables 1 and 2. Regarding the five virulence genes associated to APEC (Fig 1); 81%, 78%, 75%, 72% and 72% of the 32 *E*. *coli* strains yielded amplicons for *iss*, *ompT*, *iutA*, *iroN* and *hlyF*, respectively (Table 1). The prevalence of these genes was higher in APEC isolates (91% of the APEC strains harboured all of the mentioned genes), when compared to the AFEC isolates. In general, the presence of virulence-associated genes in AFEC strains was low, with 80% of the strains having from zero to three of the previously mentioned virulence genes (Table 1). According to the number of virulence-associated genes, 34% of the isolates were considered ExPEC.

### Statistical analysis

Significant differences (*P* = 0.035) were observed in the number of virulence-associated genes (*hlyF*, *astA*, *iroN*, *iutA* and *ompT*) found between APEC (mean, 9.2; range 5 to 16) and AFEC (mean, 6.7; range 3 to 13) isolates (Table 2). Statistical difference was found also comparing the virulence-associated genes of the isolates of phylogroup A for APEC and AFEC (*P* = 0.013; virulence score 7.2 vs 4.2). The strains belonging to phylogroup B2 exhibited the highest virulence score (mean, 14.5; range, 13 to 16) whereas the phylogroup A exhibited the lowest (mean, 5.8; range, 3 to 11). Additionally, significant differences were observed comparing B2 and D phylogroups (mean, 11.1; range 8 to 16) with A and B1 phylogroups (mean 7.4; range 3 to 13) (*P* = 0.008).

### Antimicrobial susceptibility testing and resistance genes

All the analyzed strains were multi-resistant (resistant to more than 3 antimicrobial families), including those isolated from healthy animals. Furthermore, 50% were resistant to more than eight antimicrobials. Susceptibility testing detected 11 strains resistant to cephalosporins (34%); six *bla*_CTX-M-14_, two *bla*_SHV-12_, two *bla*_CMY-2_ and one *bla*_SHV-2_. Two of these strains belonged to AFEC isolates. Two isolates were resistant to cefoxitin, and the resistance mechanism involved could not be determined.

In addition, 88% of the isolates were resistant to nalidixic acid and 91% to ciprofloxacin. Additionally, 91% of the strains were resistant to tetracycline, 78% to ampicillin, 69% to streptomycin, 63% to sulfamethoxazole, 59% to trimethoprim 34% to cefotaxime, 31% to ceftazidime, 19% to kanamycin, 16% to gentamicin, 13% to cefoxitin, 13% to chloramphenicol and 6% to florfenicol. No resistance to cefepime and colistin was observed among the isolates (Fig 1).

The presence of the *qnrS* gene was only confirmed in the isolate belonging to O25b:H4-B2-ST131. Finally, two of the APEC strains exhibited *qnrA* and two *qnrA* together with *aac(6’)-Ib-cr*. The genes *qnrB*, *qepA* and *oqxAB* were not found in this strain collection.

### Plasmid localisation of cephalosporin resistance genes

PCR-based replicon typing among the 11 cephalosporin resistant *E*. *coli* isolates showed that IncFIB replicon was present in all analysed isolates (Table 3). The replicons IncI1, IncN, IncK, IncY, IncP, IncFIA, IncHI1 and IncHI2 were also detected (Table 3).

**Table 3:** Identification and characterisation of the location of *bla*_CTX-M-14_, *bla*_SHV-2_, *bla*_SHV-12_, *bla*_CMY-2_ and *bla*_TEM_ among 11 cephalosporin resistant *E*. *coli* isolates.

Isolate^a^	ST	Replicons^b^	*bla* type	Plasmid^c^	Inc^d^	Plasmid size (kb)
N129	101	FIB	CTX-M-14	pST101	-	90
GN2221	131	N, FIB, K	TEM-1	pST131-1	N	60
			TEM-1	pST131-2	N	250
			CMY-2	pST131-3	K	120
N109	156	HI1, FIB, K	TEM-1	pST156-1	HI1	250
			SHV-2	pST156-2	K	120
N127b	156	HI2, FIB	TEM-1	-	-	-
			CTX-M-14	-	-	-
GN2233	165	FIB, Y, K	CTX-M-14	pST165	K, Y	90
N128	350	FIB	TEM-1	pST350-1	-	180
			CTX-M-14	pST350-2	-	90
N140	350	I1, FIB, K	SHV-12	pST350-3	-	90
N135	429	FIB, P, K	CMY-2	pST429	P, K	90
N139	533	I1, FIB	TEM-1	pST533	I1	120
			SHV-12	pST533	I1	120
N126	889	FIA, FIB, K	CTX-M-14	pST889	K	90
N125	1137	I1, FIB	CTX-M-14	pST1137	I1	95

p(ST number), plasmid location; Inc, identified replicon.

^a^ Isolates are divided in APEC (N) and AFEC (GN) strains.

^b^ Replicon identifications are based on positive amplifications from the PCR-based replicon typing method.

^c^ Plasmids were named based on the source strains sequence type and plasmid size.

^d^ In all *E*. *coli* isolates, replicons from plasmids containing the different *bla* genes were identified by PCR-positive amplification and by Southern hybridisation of the S1-digested fragments.

Plasmid-sizes varied between 90 and 120-kb, with the exception of two plasmids; pST131-2 and pST350-1 with 250 and 180-kb, respectively (Table 3). The most common incompatibility group detected by Southern blot was IncK (n = 5). Specific probe hybridization of S1-digested DNA demonstrated that isolate GN2221 harboured *bla*_CMY-2_ gene in a plasmid containing replicon IncK (pST131-3). The isolate N135 harbouring also *bla*_CMY-2_ gene in a 90-kb plasmid contained IncK and IncP replicons. Isolate GN2233 yielded *bla*_CTX-M-14_ in a 90-kb plasmid with two replicons (IncK and IncY). The presence of *bla*_SHV-12_ was confirmed in two isolates having a 120 and 90-kb plasmids of the IncI1 and unknown replicon family, respectively. N109 harboured *bla*_SHV-2_ in an IncK plasmid (pST156-2). Finally, no plasmid location could be confirmed for the CTX-M-14 gene of isolate N127b.

## Discussion

This study has demonstrated the presence of different clones of APEC causing outbreaks of colibacillosis during the same period of time in different broiler farms from different regions of Spain. All these isolates were multirresistant, therefore therapeutic success may have been compromised, causing a serious economic burden to the broiler industry. Moreover, the AFEC strains were also resistant to critically important antimicrobials such as cephalosporins and fluoroquinolones. Several studies have demonstrated that the *E*. *coli* population of broilers are a reservoir of antimicrobial resistance genes that may be transferred by mobile genetic elements to the community via the food chain [12]. Although efforts have been implemented to reduce the use of antimicrobials in the poultry industry, these are sometimes overused in food producing animals, and particularly in broiler farms [35]. The restriction of fluoroquinolones and cephalosporins usage in livestock for human consumption, and the implementation of measures to limit the dissemination are needed [36].

This study have also demonstrated the presence of virulence genes associated to APEC strains in commensal *E*. *coli*, indicating a possible reservoir of virulence-associated genes in this population. However, the presence of the minimal predictors of APEC virulence was much higher in APEC isolates than AFEC isolates. Furthermore, the similarity of avian pathogenic strains with human pathogenic *E*. *coli* (ExPEC) based on virulence-associated genes was confirmed in this study, since a total of 34% of the isolates could be considered ExPEC. As also suggested by other studies, certain APEC subgroups, specifically a large proportion of phylogroup A isolates, may be considered potential zoonotic agents [6].

The information provided by different typing methods is usually unrelated. MLST results, phylogenetic groups and serotyping showed similarities between strains that with PFGE were not significant. For instance, the two highly pathogenic strains O78:H9-C-ST23 shared 80% of identity by PFGE, as well as the two strains O5:H10-A-ST93, which shared 70% similarity. Additionally, two AFEC strains O3:H26-A-ST165 exhibited 90% similarity by PFGE. In these cases, the virulence gene content and the results of the susceptibility testing were equal or almost equal. However, the combination of all these techniques may be useful to discriminate between strains causing outbreaks and those with potential risk to cause disease in animals or humans. For instance, combining serotyping, phylotyping and MLST the strain GN-2221 (O25b:H4-B2-ST131) could be identified as a potential zoonotic agent causing infections in humans. Still, further studies based in animal models are necessary to properly confirm the pathogenicity of the strain.

All the serotypes found within this strain collection have been well described in previous studies [8, 16, 37–39]. In contrast, different results have been observed from the ST data, where several ST types (ST10, ST23, ST48, ST57, ST93, ST117, ST131, ST156, ST350, ST429 and ST648) have been previously associated to APEC strains [8, 16, 40–42], whereas other STs (ST101, ST165, ST189, ST297, ST533, ST539, ST624, ST650, ST889 and ST3161) have not been related to infections cause by APEC before.

The phylogroup B2 is known to harbour many more virulence-encoding genes than the rest of the *E*. *coli* phylogroups [10]. In our study, the two B2 strains found were O25b:H4-B2-ST131, which previously has been associated to human infections [43], and O2:H1-B2-ST429 which is frequently associated with poultry disease [44]. They exhibited 14 and 16 of the 29 virulence genes tested, respectively. Additionally, both strains were resistant to cephalosporins by production of CMY-2. The typical highly pathogenic APEC clonal group O78-H9-C-ST23 was found twice in this strain collection. Studies on sequencing and phylogenetic analysis of this clonal line revealed that O78 is more closely related to human strains (i. e. ST23 enterotoxigenic *E*. *coli* (ETEC)) than other APEC strains [44]. These results suggest that the *E*. *coli* population of broilers may be a potential reservoir of virulence-associated genes that could be transferred to humans through the food chain.

Our results are in line with the most commonly described ESBLs and AmpC producing *E*. *coli* in poultry production, which are CTX-M-14, CTX-M-1, CMY-2 and SHV-12 [45]. Also, these data provided evidence to the known genetic heterogeneity among ESBL-harboring *E*. *coli* isolates in broilers. In the last years, an increase in the presence of *E*. *coli* O25b:H4-B2-ST131 producing CTX-M-15 with a high virulence potential has been reported in human infections [46–48]. The high prevalence of this clonal group among multi-drug resistant isolates has important clinical and public health implications, due to the risk of treatment failure. The first time that the human ExPEC clonal group O25b:H4-B2-ST131 was detected in poultry was in Spain in 2010 [13, 49]. In both cases, the genes encoding resistance to cephalosporins were CTX-M-9. Additionally, other studies have described ST131 isolates of human origin carrying CTX-M-15 and *qnrS* in IncN plasmids [50]. It is noteworthy that our study describes for the first time a poultry *E*. *coli* isolate clonal group O25b:H4-B2-ST131 producing CMY-2 with co-resistance to flouroquinolones (*qnrS*). Interestingly, this isolate presented an exceptional phenotype, since it was resistant to fluoroquinolones (MIC = 0.25) but was susceptible to nalidixic acid (MIC = 4) according to epidemiological cut-off values. This isolate was isolated from a healthy animal, corroborating the true zoonotic potential.

Up to date, the most common plasmids carrying cephalosporin resistance genes in *E*. *coli* isolated in poultry farms belong to the IncI1, IncFIB and IncN families [51]. However, in this study, cephalosporin resistance genes are mostly associated to IncK plasmids. Moreover, in some occasions two replicas of the same ESBL gene were harboured in two different plasmids. Interestingly, isolate O25b:H4-B2-ST131 harboured two plasmids from the same incompatibility group (IncN), in the same host cell. Therefore, it is probable that another unknown replicon is present and expressed in at least one of these plasmids.

In conclusion, this study demonstrated a very diverse population of multi-drug resistant *E*. *coli* in broiler farms containing a high number of virulence-associated genes. This is probably the combination of virulence and resistance genes transferring from one strain to another via mobile genetic elements creating a multi-clonal scenario, together with *E*. *coli* strains acquiring the genes and becoming clonally successful. More epidemiological studies are necessary to identify clonal groups and resistance mechanisms with potential relevance to public health. Additionally, prudent use of antimicrobials in animal production should be implemented to reduce the burden of resistance organisms entering the food chain.
